# Human Respiratory Syncytial Virus Subgroup A and B Infections in Nasal, Bronchial, Small-Airway, and Organoid-Derived Respiratory Cultures

**DOI:** 10.1128/mSphere.00237-21

**Published:** 2021-05-12

**Authors:** L. C. Rijsbergen, M. M. Lamers, A. D. Comvalius, R. W. Koutstaal, D. Schipper, W. P. Duprex, B. L. Haagmans, R. D. de Vries, R. L. de Swart

**Affiliations:** a Department of Viroscience, Postgraduate School of Molecular Medicine, Erasmus MC, Rotterdam, the Netherlands; b Center for Vaccine Research, School of Medicine, University of Pittsburgh, Pittsburgh, Pennsylvania, USA; University of Kentucky College of Medicine

**Keywords:** air-liquid interface, airway organoids, cytokines, human airway epithelial cells, interferons, respiratory syncytial virus

## Abstract

Human respiratory syncytial virus (HRSV) is the leading cause of bronchiolitis in infants. Two subgroups of HRSV (A and B) routinely cocirculate. Most research has been performed with HRSV-A strains because these are easier to culture than HRSV-B strains. In this study, we aimed to compare the replicative fitness and HRSV-induced innate cytokine responses of HRSV-A and HRSV-B strains in disease-relevant cell culture models. We used two recombinant (r) clinical isolate-based HRSV strains (A11 and B05) and one recombinant laboratory-adapted HRSV strain (A2) to infect commercially available nasal, bronchial, and small-airway cultures. Epithelial cells from all anatomical locations were susceptible to HRSV infection despite the induction of a dominant type III interferon response. Subgroup A viruses disseminated and replicated faster than the subgroup B virus. Additionally, we studied HRSV infection and innate responses in airway organoids (AOs) cultured at air-liquid interface (ALI). Results were similar to the commercially obtained bronchial cells. In summary, we show that HRSV replicates well in cells from both the upper and the lower airways, with a slight replicative advantage for subgroup A viruses. Lastly, we showed that AOs cultured at ALI are a valuable model for studying HRSV *ex vivo* and that they can be used in the future to study factors that influence HRSV disease severity.

**IMPORTANCE** Human respiratory syncytial virus (HRSV) is the major cause of bronchiolitis and pneumonia in young infants and causes almost 200,000 deaths per year. Currently, there is no vaccine or treatment available, only a prophylactic monoclonal antibody (palivizumab). An important question in HRSV pathogenesis research is why only a fraction (1 to 3%) of infants develop severe disease. Model systems comprising disease-relevant HRSV isolates and accurate and reproducible cell culture models are indispensable to study infection, replication, and innate immune responses. Here, we used differentiated AOs cultured at ALI to model the human airways. Subgroup A viruses replicated better than subgroup B viruses, which we speculate fits with epidemiological findings that subgroup A viruses cause more severe disease in infants. By using AOs cultured at ALI, we present a highly relevant, robust, and reproducible model that allows for future studies into what drives severe HRSV disease.

## Abstract (precis)

Human respiratory syncytial virus (HRSV) is the major cause of bronchiolitis and pneumonia in young infants and causes almost 200,000 deaths per year. Currently, there is no vaccine or treatment available, only a prophylactic monoclonal antibody (palivizumab).

## INTRODUCTION

Human respiratory syncytial virus (HRSV) causes severe acute respiratory infections in infants, the elderly, and immunocompromised individuals (1). There are two antigenic subgroups of HRSV, HRSV-A and HRSV-B. Most infants experience a primary HRSV infection in their first 2 years of life (1, 2). In 15 to 50% of the cases, this results in a lower respiratory tract infection (LRTI) such as bronchiolitis or pneumonia, leading to hospitalization in 1 to 3% of the infants (3). The burden of HRSV is substantial in the elderly as well; at least 10% of winter hospitalizations are due to HRSV, with a case fatality rate of almost 10% (4). HRSV infection does not lead to protective immunity, and reinfections can occur throughout life (5, 6). Treatment options for HRSV are limited. Palivizumab is a humanized monoclonal antibody specific for the HRSV fusion (F) glycoprotein and is used as prophylaxis but is expensive and only given to infants at high risk (7, 8). Recently, another humanized monoclonal antibody against HRSV was developed (nirsevimab) (9). Ribavirin, a nucleoside analogue, is an approved drug for treating HRSV infection, but its effectiveness is disputed (10). Despite many efforts to develop an HRSV vaccine, none have been licensed (11).

HRSV is predominantly transmitted via direct contact or large droplets and targets epithelial cells in the respiratory tract (12). HRSV replication and the ensuing host inflammatory response can lead to damage of the epithelial layer. Exposure usually results in an upper respiratory tract infection (URTI), and symptoms include congestion, sneezing, and rhinorrhea (2, 13). In some cases, HRSV spreads to the lower respiratory tract and infects ciliated epithelial cells of the bronchi and terminal bronchioles (14, 15). Subsequent epithelial damage, combined with an influx of white blood cells and mucus production, can lead to mucus plugging and occlusion of the airway lumina. This results in airway obstruction and air trapping, causing apnea, difficulty breathing, and wheezing. This effect is most pronounced in infants, where the airways are still exceptionally small (13, 14). Complications in adults are associated with acute bronchitis, pneumonia, and exacerbations of chronic obstructive pulmonary disease or asthma. Where severe HRSV disease in infants is often associated with immune hyperresponsiveness, in adults, it is associated with hyporesponsiveness, which could be caused by compromised or waning immunity (1, 2).

We only have a rudimentary understanding of the development of severe HRSV disease. This is partly due to the fact that many studies have been performed in model systems that do not accurately reflect the *in vivo* situation. Immortalized cell lines like HEp-2, A549, BEAS-2B, and Vero cells are frequently used. However, these cells poorly reflect the natural target cells for HRSV and potentially do no not express the relevant cellular entry receptors. Studies in these immortalized cell lines can lead to spurious observations on entry, dissemination, and infectivity (16, 17).

Well-differentiated (wd) primary human airway epithelial models are an attractive cell culture model to study respiratory virus-host interactions. These primary human airway cultures are differentiated at the air-liquid interface (ALI) to polarized epithelial cell cultures that mimic the human respiratory tract and include the natural target cell for HRSV, ciliated epithelial cells (12, 17–20). A more recently developed model system to study respiratory virus-host interactions is based on airway organoids (AOs) (21). AOs are stem cell based, meaning that they have self-renewing capacities and thus offer an unlimited supply of cells, increasing experimental reproducibility. HRSV infection of differentiated AOs grown in Matrigel led to similar phenomena as observed in infants *in vivo*, such as swelling, detachment, and sloughing of cells into the lumen (22–24). AOs cultured at ALI can be differentiated into a pseudostratified epithelium that includes polarized ciliated epithelial cells and can also be used as a model for HRSV studies (25).

Two HRSV subgroups, HRSV-A and HRSV-B, cocirculate globally. Although both can cause severe HRSV disease, HRSV-A strains are typically associated with more severe disease than HRSV-B strains (26). Additionally, most HRSV studies are performed with subgroup A strains because these are easier to propagate than HRSV-B strains (16). Our aim in this study was to compare the replicative fitness and HRSV-induced innate cytokine responses of HRSV-A and HRSV-B strains in disease-relevant cell culture models. We used a recombinant (r) HRSV-A and an HRSV-B strain, both based on clinical isolates [rHRSV^A11^EGFP(5) (27), and rHRSV^B05^EGFP(5) (28) or rHRSV^B05^dTom(5) (27), referred to rHRSV^A11^ and rHRSV^B05^], and a laboratory-adapted rHRSV-A [rHRSV^A2^EGFP(5), referred to as rHRSV^A2^] to infect nasal, bronchial, and small-airway cultures obtained from Epithelix. All viruses expressed enhanced green fluorescent protein (EGFP) or dTomato (dTom) as a reporter protein from the 5th position in the genome, facilitating sensitive detection of HRSV-infected cells in the absence of visible cytopathic changes. We found nasal, bronchial, or small-airway cultures (SACs) were all susceptible to HRSV infection, despite induction of a type III interferon (IFN) response. This was recapitulated in AOs grown at ALI that resembled the bronchial cultures. Subgroup A viruses replicated to higher titers of cell-free virus than the subgroup B virus in all our cultures at 3 days postinfection (DPI), but the clinical isolates resulted in more infected cells (measured as percentage of virus-positive area) at 2 DPI. HRSV infection led to visible cytopathic effects, such as rounding of ciliated cells, cilia disruption, shorter cilia, and formation of small syncytia. Viral loads correlated with the production of innate cytokines, dominated by type III IFN.

## RESULTS

### Replication kinetics in well-differentiated nasal, bronchial, and small-airway cultures.

**FIG 1:**
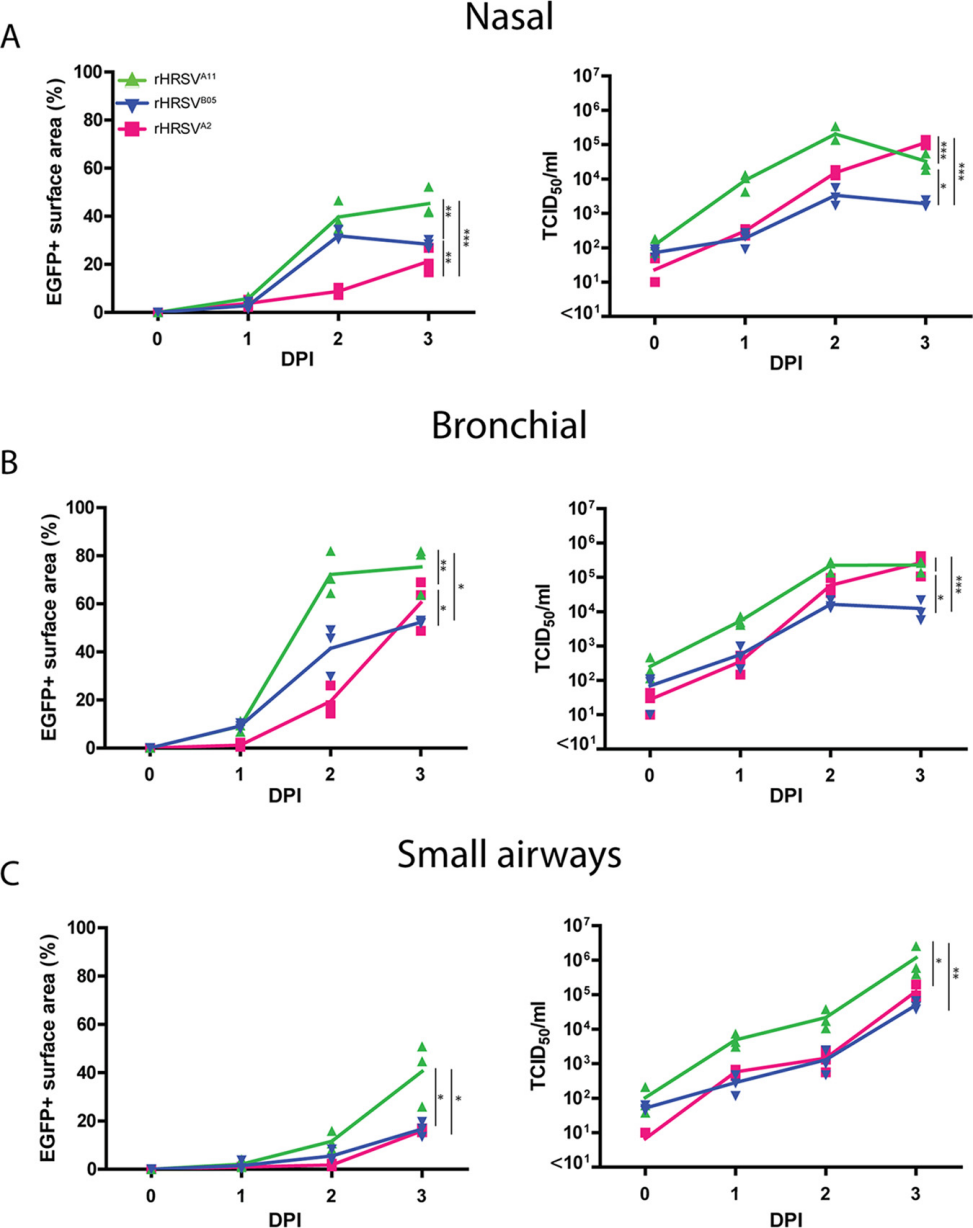
Replication kinetics of rHRSV^A2^EGFP(5), rHRSV^A11^EGFP(5), and rHRSV^B05^EGFP(5) in nasal, bronchial, and small-airway cultures. Nasal cells (A), bronchial cells (B), and small-airway cells (C) were infected with rHRSV^A2^EGFP(5), rHRSV^A11^EGFP(5), or rHRSV^B05^EGFP(5) at a standardized low viral inoculum. The percentage of EGFP^+^ surface area was determined by confocal microscopy, and the viral titers were determined by endpoint titrations of apical washes (TCID_50_/ml). One independent experiment is shown, and all experiments were performed in triplicate. Differences between the growth curves were statistically analyzed by two-way ANOVA (*, *P* = 0.05; **, *P* = 0.01; ***, *P* = 0.001; ****, *P* < 0.0001). Mean and individual replicates are shown.

Well-differentiated airway cultures from the nose, bronchus, and SACs (bronchioles) were purchased from Epithelix and infected with a relatively low inoculum of rHRSV^A2^ (laboratory adapted), rHRSV^A11^, and rHRSV^B05^ (clinical isolate based). After a single round of replication (24 h), we detected slightly more foci of HRSV infection in nasal and bronchial cells than in the SACs (Fig. S1 in the supplemental material). This shows that with a standardized inoculum and single replication cycle, differences in susceptibility and permissiveness between the cells could already be shown. Further dissemination was studied by measuring the production of cell-free virus and the surface area of HRSV-infected cells based on fluorescence over time (Fig. 1). Both rHRSV-A strains replicated to significantly higher cell-free viral titers (±10^5^ to 10^6^ 50% tissue culture infective dose [TCID_50_]/ml) than the rHRSV-B strain (10^4^ TCID_50_/ml) over time, especially in nasal and bronchial cultures. However, the percentage of EGFP-positive (EGFP^+^) surface area at 1 and 2 DPI was significantly higher for both clinical isolate-based strains than for the laboratory-adapted strain. The clinical-based rHRSV^A11^ virus disseminated to a higher infected surface area than the clinical-based rHRSV^B05^.

10.1128/mSphere.00237-21.1 FIG S1 Number of fluorescent foci after 24 h in nasal, bronchial, small-airway cultures (SACs), and airway organoids (AOs) cultured at the air-liquid-interface (ALI) 24 h after infection. Nasal cells, bronchial cells, SACs, and AOs cultured at ALI were infected with rHRSV^A2^EGFP(5), rHRSV^A11^EGFP(5), or rHRSV^B05^EGFP(5) at a standardized low inoculum. The amount of EGFP^+^ foci was determined by confocal microscopy and quantified with Fiji. One independent experiment is shown, and all experiments were performed in triplicate. Differences between the growth curves were statistically analyzed by two-way ANOVA (*, *P* = 0.05; **, *P* = 0.01; ***, *P* = 0.001; ****, *P* < 0.0001). Mean and standard deviation are shown. Download FIG S1, TIF file, 0.4 MB. Copyright © 2021 Rijsbergen et al. 2021 Rijsbergen et al. https://creativecommons.org/licenses/by/4.0/ This content is distributed under the terms of the Creative Commons Attribution 4.0 International license.

### Cytopathology in well-differentiated nasal, bronchial, and small-airway cultures.

**FIG 2:**
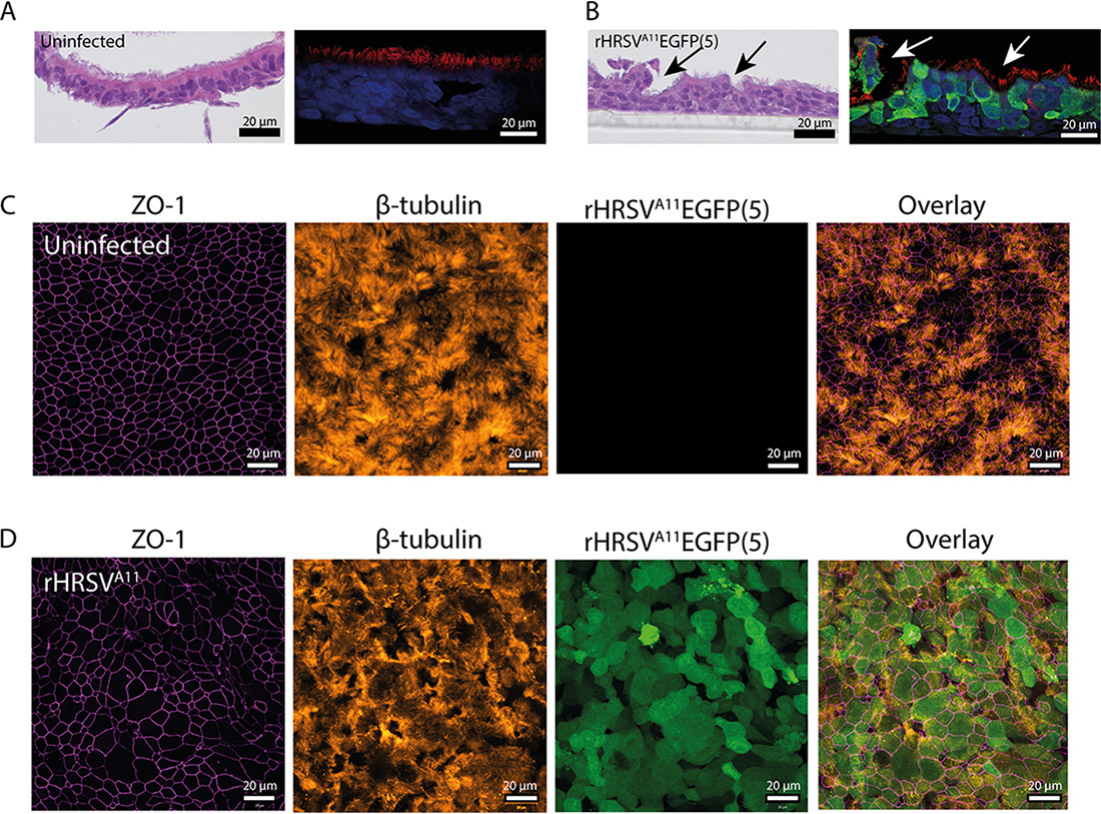
Immunohistochemistry and indirect immunofluorescence on bronchial cells infected with HRSV. Bronchial cells were infected with either rHRSV^A2^EGFP(5), rHRSV^A11^EGFP(5), or rHRSV^B05^EGFP(5) at a standardized low viral inoculum. Samples were fixed in formalin at 3 days postinfection (DPI) and embedded in paraffin. (A and B) Paraffin-embedded slides were used for H&E staining and indirect immunofluorescence using antibodies against green fluorescent protein (HRSV, green), acetylated α-tubulin (cilia, red), and Hoechst (nuclei, blue). Arrows indicate putative mucus channels in the cultures. (C and D) Transwell filters were additionally stained with antibodies against zona-occludens 1 (tight junctions, magenta) and acetylated α-tubulin (cilia, orange). Representative images are shown of rHRSV^A11^EGFP(5).

To assess the tropism of HRSV and the associated cytopathic effect (CPE) in primary cultures, we performed a histological analysis of airway cultures at 3 DPI, in combination with indirect immunofluorescence staining (IIF). The uninfected nasal and bronchial cultures consisted of a pseudostratified epithelium, and ciliated cells were abundantly present at the apical side (Fig. 2A and C [bronchial]; Fig. S2A and C [nasal]). In the HRSV-infected nasal and bronchial cultures, infected cells were mainly present at the apical side of the epithelium and were predominantly ciliated epithelial cells. We observed hyperplasia of HRSV-infected cells and the formation of small channels in the culture (Fig. 2A and B; Fig. S2A and B). Microscopically, we observed an increase in mucus production, and cilia actively transported the mucus through these channels (data not shown). These alterations were not observed in the uninfected cultures. Furthermore, we observed cilia degeneration (shortened and damaged cilia, rounding of ciliated cells) and damaged cell layer, the latter visualized by the loss of tight junctions caused by small syncytium formation, in both nasal and bronchial cultures (Fig. 2C and D; Fig. S2C and D). Differences in CPE between rHRSV-A and rHRSV-B strains were not observed. Compared to nasal and bronchial cultures, uninfected SACs contained a thinner basal cell layer and fewer ciliated epithelial cells (Fig. S3A and C). In SACs, HRSV also mainly targeted ciliated epithelial cells (Fig. S3B and D), but the infection was more focal than the nasal and bronchial cultures. We observed infected cell hyperplasia compared to the uninfected control throughout the epithelial layer, but cilia and tight junctions remained intact (Fig. S3A and B).

10.1128/mSphere.00237-21.2 FIG S2 Immunohistochemistry and indirect immunofluorescence on HRSV-infected nasal cells. Nasal cells were infected with either rHRSV^A2^EGFP(5), rHRSV^A11^EGFP(5), or rHRSV^B05^EGFP(5) at a standardized low viral inoculum. Samples were fixed in formalin at 3 DPI and embedded in paraffin. (A and B) Paraffin-embedded slides were used for H&E staining and indirect immunofluorescence using antibodies against green fluorescent protein (HRSV, green), acetylated α-tubulin (cilia, red), and Hoechst (nuclei, blue). (C and D) Transwell filters were additionally stained with antibodies against zona-occludens 1 (tight junctions, magenta) and acetylated α-tubulin (cilia, orange). Representative images are shown of rHRSV^A11^. Download FIG S2, TIF file, 2.6 MB. Copyright © 2021 Rijsbergen et al. 2021 Rijsbergen et al. https://creativecommons.org/licenses/by/4.0/ This content is distributed under the terms of the Creative Commons Attribution 4.0 International license.

10.1128/mSphere.00237-21.3 FIG S3 Immunohistochemistry and indirect immunofluorescence on HRSV-infected small-airway cells. Small-airway cultures were infected with either rHRSV^A2^EGFP(5), rHRSV^A11^EGFP(5), or rHRSV^B05^EGFP(5) at a standardized low viral inoculum. Samples were fixed in formalin at 3 days postinfection (DPI) and embedded in paraffin. (A and B) Paraffin-embedded slides were used for H&E staining and indirect immunofluorescence using antibodies against green fluorescent protein (HRSV, green), acetylated α-tubulin (cilia, red), and Hoechst (nuclei, blue). (C and D) Transwell filters were additionally stained with antibodies against zona-occludens 1 (tight junctions, magenta) and acetylated α-tubulin (cilia, orange). Representative images are shown of rHRSV^A11^. Download FIG S3, TIF file, 2.6 MB. Copyright © 2021 Rijsbergen et al. 2021 Rijsbergen et al. https://creativecommons.org/licenses/by/4.0/ This content is distributed under the terms of the Creative Commons Attribution 4.0 International license.

### Cytokine responses in well-differentiated nasal, bronchial, and small-airway cultures.

**FIG 3:**
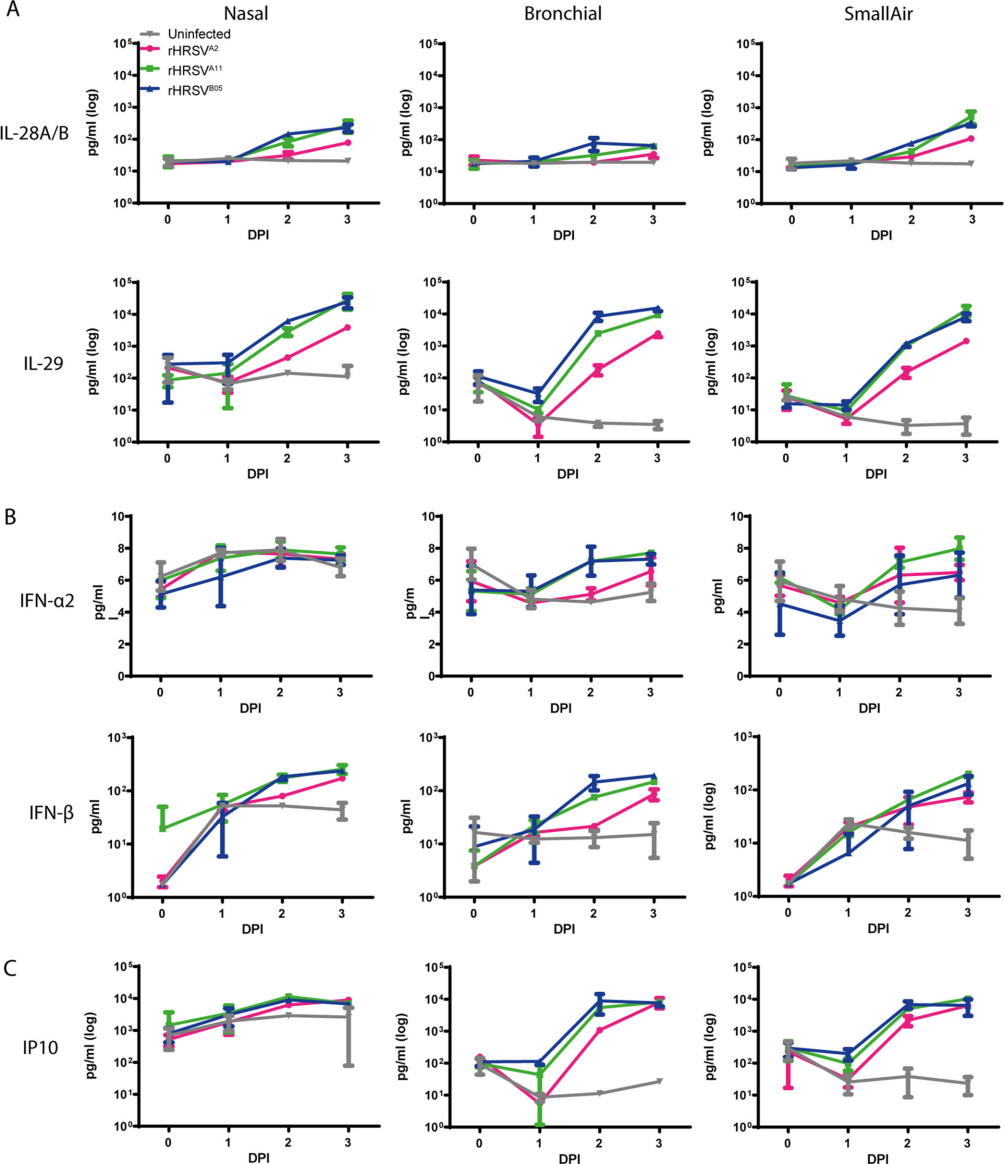
Type III IFN, type I IFN, and IP10 production in HRSV-infected nasal, bronchial, and small-airway cells. Nasal, bronchial, and small-airway cells were infected with either rHRSV^A2^EGFP(5), rHRSV^A11^EGFP(5), or rHRSV^B05^EGFP(5) at standardized low viral inoculum. Apical washes were taken at 0, 1, 2, and 3 days postinfection (DPI), and type III IFN (IL28A/B, IL-29) (A), type I IFN (IFN-α2 and IFN-β) (B), and IP10 (C) were quantified using the BD LEGENDplex human antivirus response panel. One independent experiment is shown, and all experiments were performed in triplicate. Mean and standard deviation are shown.

To evaluate the antiviral response of primary differentiated epithelial cells to HRSV infection, we harvested apical and basolateral washes at 1, 2, and 3 DPI and measured cytokine levels. Cytokines were predominantly detected in the apical washes. Type III interferons (IFN) (interleukin 28 A and B [IL-28A/B] and IL-29) were the main cytokines produced in all airway cultures. IL-29 increased at least 100-fold compared to uninfected cultures. IL-28A/B also increased 10- to 100-fold over time and was most abundant in the nasal cultures and SACs (Fig. 3A). Type I IFNs (IFN-α2 and IFN-β) were produced to a lesser extent than type III IFN. The concentration of IFN-α2 hardly increased after infection compared to uninfected controls, and IFN-β was 2- to 3-fold elevated in all cultures compared to uninfected controls (Fig. 3B). Overall, both rHRSV^A11^ and rHRSV^B05^ induced higher levels of IFNs at 2 DPI than rHRSV^A2^, which is in accordance with the percentages of EGFP^+^ surface area (Fig. 1). Notably, the interferon response clearly lagged behind the viral replication, and viral titers correlated positively with interferon production (Fig. S4). Early-stage inflammatory cytokine IP10 was increased in all the HRSV-infected airway cultures and also correlated positively with viral replication (Fig. 3C; Fig. S4). We also measured IL-6, IL-1b, tumor necrosis factor alpha (TNF-α), IFN-γ, IL-8, IL12p70, granulocyte-monocyte colony-stimulating factor (GM-CSF), and IL-10. These cytokines were produced in low quantities after HRSV infection, with the exception of IL-6. This cytokine was more upregulated after infection with the two clinical-based isolates compared to the laboratory-adapted strain in nasal cultures and SACs (Fig. S5). Cytokines were also measured in the basolateral compartments, but in general, low levels of cytokines were detected, with the exception of IL-6 (data not shown).

10.1128/mSphere.00237-21.4 FIG S4 Type III IFN, type I IFN, and IP10 correlated with the viral load in nasal, bronchial, and small-airway cells. Nasal, bronchial, and small-airway cells were infected with either rHRSV^A2^EGFP(5), rHRSV^A11^EGFP(5), or rHRSV^B05^EGFP(5) at a standardized low viral inoculum. The log-transformed viral loads were correlated with the log-transformed levels of type III IFN (IL28A/B, IL29), type I IFN (IFN-α2 and IFN-β), and IP10 using the Pearson correlation coefficient. Download FIG S4, TIF file, 1.3 MB. Copyright © 2021 Rijsbergen et al. 2021 Rijsbergen et al. https://creativecommons.org/licenses/by/4.0/ This content is distributed under the terms of the Creative Commons Attribution 4.0 International license.

10.1128/mSphere.00237-21.5 FIG S5 Cytokine production in HRSV-infected nasal, bronchial, and small-airway cells. Nasal, bronchial, and small-airway cells were infected with either rHRSV^A2^EGFP(5), rHRSV^A11^EGFP(5), or rHRSV^B05^EGFP(5) at a standardized low viral inoculum. Apical supernatants were taken at 1, 2, and 3 days postinfection (DPI), and IL-6, IL-1β, TNF-α, IFN-γ, IL-8, IL12p70, GM-CSF, and IL-10 were quantified using the LEGENDplex human antivirus response panel. One independent experiment is shown, and all experiments were performed in triplicate. Mean and standard deviation are shown. Download FIG S5, TIF file, 1.2 MB. Copyright © 2021 Rijsbergen et al. 2021 Rijsbergen et al. https://creativecommons.org/licenses/by/4.0/ This content is distributed under the terms of the Creative Commons Attribution 4.0 International license.

### HRSV replication kinetics in well-differentiated airway organoid cultures at ALI.

**FIG 4:**
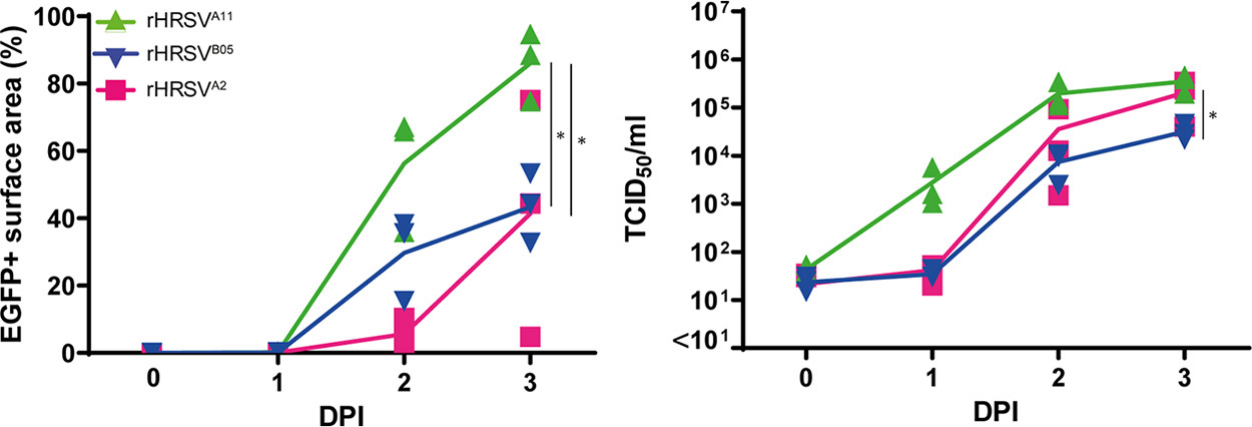
Replication kinetics of rHRSV^A2^EGFP(5), rHRSV^A11^EGFP(5), and rHRSV^B05^EGFP(5) in primary well-differentiated airway organoid cultures at ALI. Primary well-differentiated airway organoid cultures at ALI were infected with rHRSV^A2^EGFP(5), rHRSV^A11^EGFP(5), or rHRSV^B05^EGFP(5) at a standardized low viral inoculum. The percentage EGFP^+^ surface area was determined by confocal laser scanning microscopy, and the viral titers were determined by endpoint titrations of the apical washes (TCID_50_/ml). One representative experiment is shown that was performed in triplicate. Differences between the growth curves were statistically analyzed with a two-way ANOVA (*, *P* = 0.05). Mean and individual replicates are shown.

In addition to studying HRSV replication kinetics and innate immune responses in commercially available primary cells, we also studied these in AOs cultured at ALI as described before (25). These AOs were obtained from adult human lung stem cells from the distal airways and cultured at ALI for 4 weeks to allow differentiation of the cells into pseudostratified epithelium, including ciliated epithelial cells. Cells were infected with a low standardized inoculum of rHRSV, and the replication kinetics, dissemination, cytopathology, and innate cytokine responses were assessed. In general, HRSV replication kinetics were comparable with the commercially available bronchial cultures: infection with the clinical isolate-based viruses resulted in a higher percentage virus-infected surface area (especially at 2 DPI), and infection with rHRSV-A strains resulted in higher viral titers over time (Fig. 4; Fig. S1). rHRSV^A11^ disseminated better than the other viruses, resulting in significantly more infected cells.

### Cytopathology in well-differentiated airway organoid cultures at ALI.

**FIG 5:**
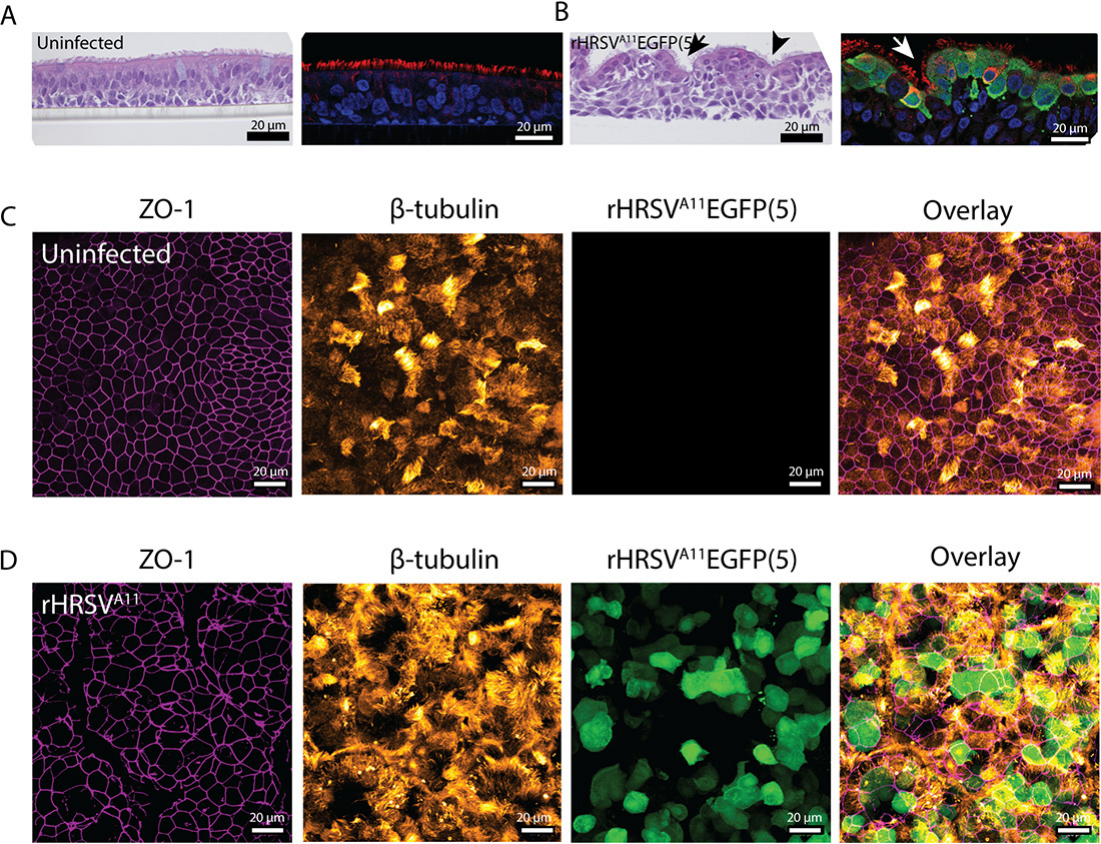
Immunohistochemistry and indirect immunofluorescence on primary well-differentiated airway organoid cultures at ALI infected with HRSV. Well-differentiated airway organoid cultures at ALI were infected with either rHRSV^A2^EGFP(5), rHRSV^A11^EGFP(5), or rHRSV^B05^EGFP(5) at a standardized low viral inoculum. Samples were fixed in formalin at 3 days postinfection (DPI) and embedded in paraffin. (A and B) Paraffin-embedded slides were used for H&E staining and indirect immunofluorescence using antibodies against green fluorescent protein (HRSV, green), acetylated α-tubulin (cilia, red), and Hoechst (nuclei, blue). Arrows indicate putative mucus channels in the cultures. (C and D) Transwell filters were additionally stained with antibodies against zona-occludens 1 (tight junctions, magenta) and acetylated α-tubulin (cilia, orange). Representative images are shown of rRHRSV^A11^.

To assess the tropism of HRSV and the associated CPE, we used AOs cultured at ALI. First, we created organoids (spheroids in Matrigel) from human bronchial tissue obtained after lung resection surgery. We cultured these undifferentiated organoids in Matrigel for 10 to 14 days and then dissociated them into single cells. These cells were seeded on Transwell membranes and grown at ALI for 4 weeks, allowing differentiation. We characterized the cultures by hematoxylin and eosin (H&E) and IIF staining at 3 DPI. The uninfected cultures had multiple cell layers and an abundance of ciliated cells (Fig. 5A and C). HRSV infected mainly ciliated epithelial cells. Degenerated cilia, rounding of infected cells, and small syncytia were observed in infected cultures compared to uninfected controls, and the integrity of the tight junctions was affected by HRSV infection (Fig. 5B and D). We observed an increase in mucus production and the formation of small channels (Fig. 5A and B), similar to the commercially available nasal and bronchial cultures (Fig. 2A and B; Fig. S1A and B). Differences in CPE between rHRSV-A and rHRSV-B strains were not observed.

### Cytokine production in well-differentiated airway organoid cultures at ALI.

**FIG 6:**
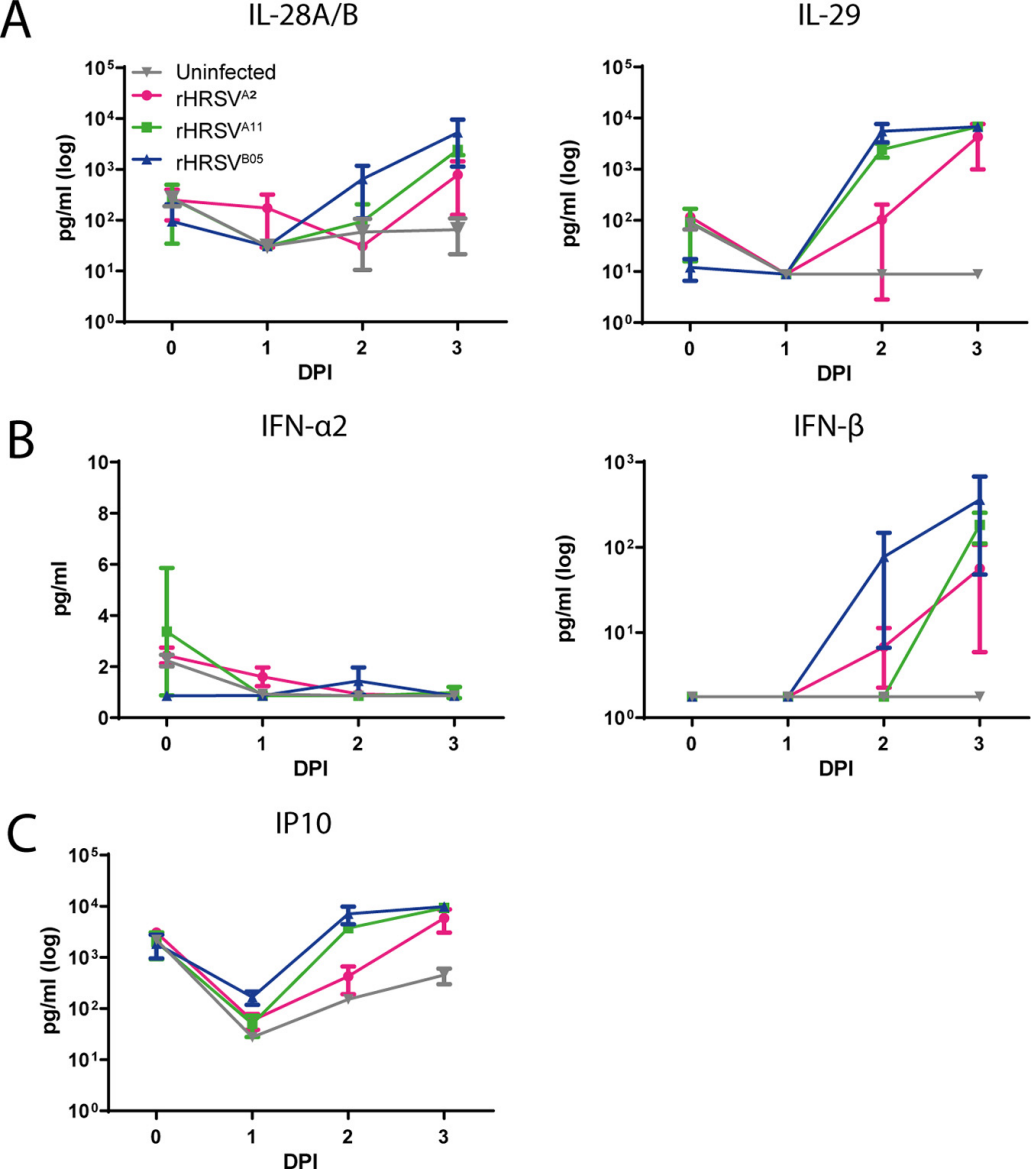
Type III IFN, type I IFN, and IP10 production in HRSV-infected primary well-differentiated airway organoid cultures at ALI. Well-differentiated airway organoid cultures at ALI were infected with either rHRSV^A2^EGFP(5), rHRSV^A11^EGFP(5), or rHRSV^B05^EGFP(5) at a standardized low viral inoculum. Apical washes were taken at 0, 1, 2, and 3 days postinfection (DPI), and type III IFN (IL28A/B, IL-29) (A), type I IFN (IFN-α2 and IFN-β) (B), and IP10 (C) were quantified using the BD LEGENDplex human antivirus response panel. One independent experiment is shown, and all experiments were performed in triplicate. Mean and standard deviation are shown.

Next, we harvested culture supernatant from the apical side at 0, 1, 2, and 3 DPI and measured the same cytokines as measured in commercially obtained nasal, bronchial, and small-airway cells (Fig. 6; Fig. S5). We observed an increase in type III IFNs; IL-29 increased the most upon HRSV infection, but IL-28A/B was also elevated (Fig. 6A). ALI-differentiated AOs produced IFN-α, but IFN-β production was more pronounced (Fig. 6B). General inflammatory marker IP10 was increased after HRSV infection (Fig. 6C). Type III IFN, type I IFN, and IP10 were all induced by HRSV replication, indicated by a positive correlation between cytokine levels and viral loads (Fig. S6). IL-6, IL-1b, TNF-α, IFN-γ, IL-8, IL12p70, GM-CSF, and IL-10 were produced in small amounts (or not at all) in AOs after rHRSV infection (Fig. S7). Overall, cytokine profiles were similar to the cytokine profiles in the commercially purchased bronchial epithelial cells; only IL-28A/B was less produced in these cultures.

10.1128/mSphere.00237-21.6 FIG S6 Type III IFN, type I IFN, and IP10 correlated with the viral load in primary well-differentiated airway organoid cultures at ALI. Well-differentiated airway organoid cultures at ALI were infected with either rHRSV^A2^EGFP(5), rHRSV^A11^EGFP(5), or rHRSV^B05^EGFP(5) at a standardized low viral inoculum. The log-transformed viral loads were correlated with the log-transformed levels of type III IFN (IL28A/B, IL29), type I IFN (IFN-α2 and IFN-β), and IP10 using the Pearson correlation coefficient. Download FIG S6, TIF file, 0.7 MB. Copyright © 2021 Rijsbergen et al. 2021 Rijsbergen et al. https://creativecommons.org/licenses/by/4.0/ This content is distributed under the terms of the Creative Commons Attribution 4.0 International license.

10.1128/mSphere.00237-21.7 FIG S7 Cytokine production in HRSV-infected primary well-differentiated airway organoid cultures at ALI. Well-differentiated airway organoid cultures at ALI were infected with either rHRSV^A2^EGFP(5), rHRSV^A11^EGFP(5), or rHRSV^B05^EGFP(5) at a standardized low viral inoculum. Apical supernatants were taken at 1, 2, and 3 days postinfection (DPI), and IL-6, IL-1β, TNF-α, IFN-γ, IL-8, IL12p70, GM-CSF, and IL-10 were quantified using the LEGENDplex human antivirus response panel. One independent experiment is shown, and all experiments were performed in triplicate. Mean and standard deviation are shown. Download FIG S7, TIF file, 0.7 MB. Copyright © 2021 Rijsbergen et al. 2021 Rijsbergen et al. https://creativecommons.org/licenses/by/4.0/ This content is distributed under the terms of the Creative Commons Attribution 4.0 International license.

### HRSV-A and HRSV-B competition experiments in airway organoids cultures at ALI.

**FIG 7:**
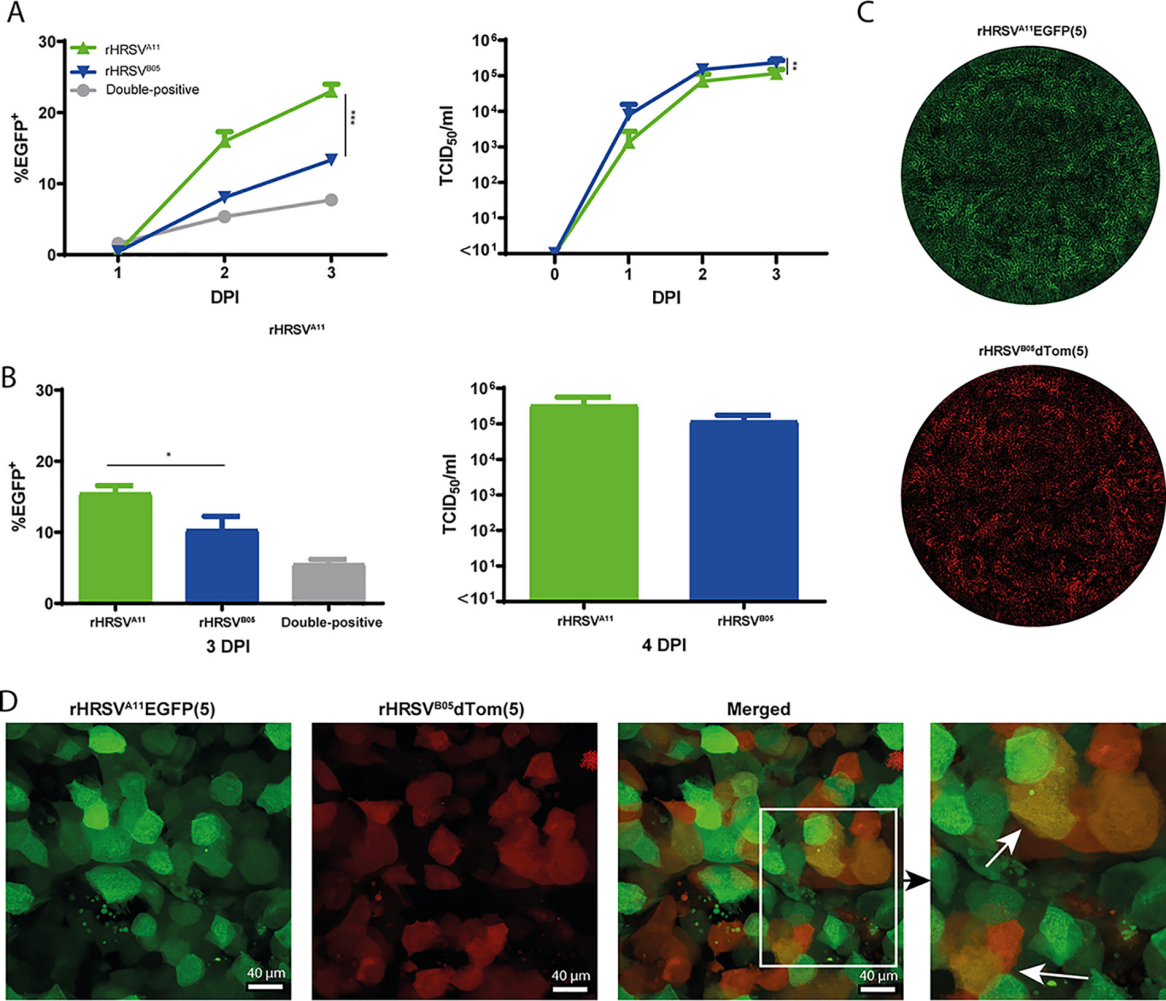
rHRSV^A11^EGFP(5) and rHRSV^B05^dTom(5) competition experiment in primary well-differentiated airway organoid cultures at ALI. Primary well-differentiated airway organoid cultures at ALI were infected with rHRSV^A11^EGFP(5) and rHRSV^B05^dTom(5) at a standardized low viral inoculum per virus and either washed apically daily (A) or not (B). The percentage of EGFP^+^ or dTom^+^ surface area was determined by confocal microscopy, and the viral titers were determined by endpoint titrations of the supernatant from the apical side (TCID_50_/ml) at 1, 2, or 3 days postinfection (DPI). The dissemination throughout the culture was imaged by using laser scanning confocal microscopy (C), and Transwell filters were excised and analyzed at high resolution to identify double-infected cells (indicated by arrows) (D). The percentages of EGFP^+^ and dTom^+^ cells were quantified by colocalization analysis in Fiji. Experiments were performed in triplicate. Differences between the growth curves were statistically analyzed with a two-way ANOVA or a Student's *t* test (*, *P* < 0.05; **, *P* < 0.01; ***, *P* < 0.005). Mean and standard deviation are shown.

In infection experiments in nasal, bronchial, SACs, and AOs, rHRSV^A11^ has an infectivity advantage over rHRSV^B05^ when comparing both production of cell-free virus and percentage EGFP^+^. To confirm this infectivity advantage of rHRSV^A11^ over rHRSV^B05^, we performed a direct competition experiment. We infected AOs cultured at ALI with rHRSV^A11^ expressing EGFP and rHRSV^B05^ expressing dTom. Both viruses were added simultaneously at the same low input, and infections were followed over time. Two different experimental setups were used, daily confocal microscopy accompanied by apical washes after infection (Fig. 7A) or daily confocal microscopy, but only performing an apical wash at 4 DPI (Fig. 7B). In both setups, we found that rHRSV^A11^ replicated to a significantly higher cell-free viral titer and disseminated to a larger virus-positive area than rHRSV^B05^. We observed both single-infected (rHRSV^A11^ or rHRSV^B05^ positive) and double-infected cells (rHRSV^A11^ and rHRSV^B05^ positive). We quantified the percentage of double-positive cells, which was about 5%. In conclusion, we observed that both viruses can spread well in the culture and were able to coinfect cells (Fig. 7A to D).

## DISCUSSION

In this study, we aimed to compare the replicative fitness and HRSV-induced innate cytokine responses of rHRSV-A and rHRSV-B strains in disease-relevant cell culture models. We used two recombinant clinical isolate-based HRSV strains, rHRSV^A11^ and rHRSV^B05^, and one recombinant laboratory-adapted HRSV strain, rHRSV^A2^, to infect commercially available nasal, bronchial, and small-airway cultures. Epithelial cells from all anatomical locations were susceptible to HRSV infection despite the induction of a robust type III interferon response. Subgroup A viruses disseminated and replicated faster than the subgroup B virus. Additionally, we studied HRSV infection and innate responses in AO cultured at ALI. The results in this model were similar to the results in the commercially obtained bronchial cells. In summary, we show that HRSV replicates well in cells from both the upper and the lower airways, with a slight infectivity advantage for subgroup A viruses. That finding fits with clinical observations that HRSV-A causes more severe disease than HRSV-B. Lastly, we showed that AOs cultured at ALI are a valuable model for studying HRSV *in vitro*. This model can be used for future experiments studying intrinsic and extrinsic factors that influence HRSV infection.

HRSV susceptibility studies with cells from different anatomical sites of the respiratory tract have rarely been performed. We found that, with a relatively low standardized inoculum, nasal and bronchial cells seemed more susceptible than SACs. However, HRSV replicated and disseminated efficiently in all the cultures over time. HRSV replication kinetics in nasal and bronchial cultures have also been shown in a previous study in which they used well-differentiated nasal and bronchial epithelial cells (29). These results match with what is observed in HRSV-infected children, where viral loads in nasal aspirates and deep tracheal aspirates correlate (30). We show that HRSV replicates in SACs. Infection of smaller bronchioles in infants can lead to mechanical obstruction due to inflammatory response, one of the hallmarks of the pathogenesis of HRSV bronchiolitis (14). The combined results of our study and the previously mentioned studies highlight that HRSV efficiently replicates and disseminates in airway cells from different anatomical locations.

In human HRSV disease, ciliated epithelial cells are the main target cell, and the infection is mostly restricted to the luminal side of the airways (14). Several studies have shown this *in vitro*, and our results in primary cells and with clinical isolate-based viruses confirm these observations (12, 17, 31–33). Additionally, we observed clear CPE at 3 DPI, such as cilia degeneration and remodeling of the epithelium. Results in literature are conflicting: cilia degeneration, such as shorter and damaged cilia, was previously described in other studies using either (pediatric) well-differentiated primary bronchial or nasal epithelial cells in combination with both clinical isolates and laboratory-adapted viruses (17, 31, 34). However, in other studies with primary well-differentiated bronchial or nasal epithelial cells, syncytium formation or cell damage were not observed (12, 34). In the latter studies, the major difference was that another recombinant HRSV strain was used (rgRSV, an HRSV-A2-derived recombinant virus), a highly laboratory-adapted strain with green fluorescent protein in the first position of the genome.

Clinical manifestations in HRSV bronchiolitis patients include epithelial cell sloughing, cell death, increased mucus production leading to mucus plugs, and occasional syncytium formation (14). We did not measure cell death or epithelial cell sloughing in our model, but we observed an increase in mucus production upon HRSV infection. We also observed alterations of the epithelium, including loss of tight junction integrity, formation of small syncytia, and formation of channels through which mucus was transported (observational data). These observations have been described in other studies using (pediatric) well-differentiated primary epithelial cells (17, 31, 35, 36). There is one study that did not describe any cytopathology in well-differentiated primary nasal epithelial cells; in this study, laboratory-adapted strains (HRSV-A2 and HRSV-A long) were used at a low multiplicity of infection and measured up to 36 h postinfection (HPI) (37).

Although infection of well-differentiated primary airway cultures with HRSV has been described, studies directly comparing laboratory-adapted strains and clinical isolates or HRSV-A versus HRSV-B are rare. Since we used recombinant HRSV strains, we were able to show that clinical isolates disseminated faster at early time points (1 and 2 DPI) than the laboratory-adapted virus, measured by surface area of infected cells. We did not observe these differences in cell-free virus titers. A limitation when comparing different virus strains in different cell types is that the susceptibility, permissiveness, and cell receptor usage can be different for each cell type (38). However, we believe that dissemination (fluorescence surface area) in combination with CPE is a better proxy for human disease severity than cell-free virus titers. Other studies have shown that HRSV-A2 replicates to higher or similar viral loads than clinical isolates, but this is based on TCID_50_/ml and not infected cells or infected surface area (17, 31, 32).

We also found that, in general, subgroup A viruses resulted in higher cell-free titers and percentage of virus-positive area than the subgroup B virus, which is in agreement with clinical observations that HRSV-A causes more severe disease (26). We also compared the infectivity of these viruses by both performing stand-alone infections and a direct competition experiment. rHRSV^A11^ always replicated and disseminated better than rHRSV^B05^, but both strains were able to disseminate and infect cells in the culture. We also found dual-infected cells in our cultures. In another study, rHRSV^A11^ expressing EGFP and rHRSV^B05^ expressing dTom were used in cotton rats and cotton rat cell lines. Occasional double-infected cells were observed, both *in vitro* and *in vivo* (27). Our data confirm these observations, with as added value that we use primary human epithelial cells. Future studies elucidating coinfections and the factors underlying the replicative advantage of HRSV-A subgroup viruses are required.

Early dissemination in the airways can be influenced by host innate immune responses. The postinfection cytokine response in our cultures was dominated by type III IFNs. Type I IFNs and IP10 were additionally produced as described previously (17, 31, 36, 37). We also measured background cytokine levels in our cultures, which is probably an inherent property of the cultures in combination with daily washing of the cells, which can cause immune activation. Nevertheless, infected cultures showed a clear increase in cytokine production. Surprisingly, the abundance of type III IFNs did not hinder HRSV replication; the innate immune response might either be too late or insufficient. Another option is that immune cells are required to clear the virus effectively. Experiments with IFNs and cocultures with innate immune cells would be better suited to study the interactions between innate cytokines and HRSV infections.

As experiments in commercial primary airway cultures are expensive and dependent on suppliers, we validated a robust model system to study HRSV infections. It has been reported that AOs are susceptible to HRSV infection and reproduce several characteristics of HRSV disease (epithelial cell shedding, mucus production) (22–24). However, these studies used AOs in a basement matrix. We decided to evaluate this model further by culturing in-house-developed AOs at ALI to create a well-differentiated stem cell-based epithelial cell model, reflecting the natural epithelial barrier in the human respiratory tract with a direct interface between air and submucosal fluids. We assessed replication kinetics of the three rHRSV strains and found that replication in AOs cultured at ALI was comparable to replication kinetics in bronchial cultures from Epithelix. Similar to observations in commercially available cells, we found that in AOs cultures at ALI, mainly ciliated cells were infected, with a loss of tight junction integrity and an increase in mucus production. Finally, we showed the cytokine response upon infection was dominated by type III IFNs. Also, IP10 was increased, which was shown previously in AO cultures in Matrigel (22). Taken together, we concluded that an AO-based well-differentiated model system accurately resembles commercially obtained bronchial cells.

In conclusion, we have shown that the combination of primary airway cultures with recombinant clinical isolate-based HRSV strains, expressing reporter proteins, is a powerful tool to study HRSV-host interactions *in vitro*. Additionally, we have refined stem cell-based cultures developed in-house for HRSV infection studies. Using these models, we demonstrated how HRSV rapidly disseminates throughout anatomically different airway cultures in the face of innate immune response. In follow-up experiments, we will use these models to investigate the influence of host factors, such as interactions with innate immune responses and bacterial and viral coinfections, on HRSV dissemination.

## MATERIALS AND METHODS

### Viruses.

Virus stocks were grown on HEp-2 cells by inoculating cell pellets at a multiplicity of infection of 0.01 for 1 to 2 h at 37°C, followed by seeding into T175 cm^2^ tissue culture flasks (for growth kinetics in HEp-2 cells, see Fig. S8 in the supplemental material). When 90 to 100% of CPE was observed, cells were scraped from the flasks and sonicated three times for 30 s, and subsequently, cell debris was removed by centrifugation for 15 min at 600 × *g*. The supernatant was mixed with sucrose to a final concentration of 25% (vol/vol) for stability, snap-frozen, and stored at −80°C. rHRSV^A2^ [rHRSV^A2^EGFP(5)] was commercially obtained from ViraTree (product no. R125), and previously rescued rHRSV^A11^ [rHRSV^A11^EGFP(5), passage 5]; rHRSV^B05^ rHRSV^B05^EGFP(5), passage 6; and rHRSV^B05^dTom(5), passage 7 have been described before (the 5 between brackets refers to the position of the additional transcriptional unit in the genome) (27, 28).

10.1128/mSphere.00237-21.8 FIG S8 Growth curves of rHRSV^A2^EGFP(5), rHRSV^A11^EGFP(5), and rHRSV^B05^EGFP(5) in HEp-2 cells. HEp-2 cells were infected with either rHRSV^A2^EGFP(5), rHRSV^A11^EGFP(5), or rHRSV^B05^EGFP(5) at a multiplicity of infection of 0.01. (A) Percentage of EGFP^+^ cells was determined by flow cytometry, and the viral titers were determined by endpoint titrations (TCID_50_/ml). Cytopathic effects was assessed by confocal microscopy. One or two independent representative experiments are shown, and all experiments were performed in triplicate. Differences between the growth curves were statistically analyzed with a two-way ANOVA (*, *P* = 0.05; **, *P* = 0.01, ***, *P* = 0.001; ****, *P* < 0.0001). Mean and standard deviation are shown. Download FIG S8, TIF file, 2.4 MB. Copyright © 2021 Rijsbergen et al. 2021 Rijsbergen et al. https://creativecommons.org/licenses/by/4.0/ This content is distributed under the terms of the Creative Commons Attribution 4.0 International license.

### Human primary airway culture and differentiation.

MucilAir (primary nasal or bronchial human airway epithelial cells) and SmallAir (primary small-airway human airway epithelial cells) were obtained from Epithelix (Epithelix Sàrl, Geneva, Switzerland) and cultured according to the manufacturer’s instructions. The culturing of human airway organoids was developed in-house, and differentiation on ALI was performed as described before (22, 25). In short, nontumor lung tissue, obtained from patients undergoing lung resection surgery for lung cancer, was used to derive adult human lung stem cells from the distal airways (Medical Ethical Committee of the Erasmus MC Rotterdam, METC 2012-512) (22). Undifferentiated AOs were cultured in Matrigel (Corning) droplets (∼30 μl) with 250 μl AO medium. To obtain differentiated organoid-derived cultures at ALI, organoids were made into single cells with TrypLE Express and consequently seeded on Transwell membranes (Corning) coated with rat tail collagen type I (Fisher Scientific). The single AO cells were seeded in AO medium and complete base medium (CBM; Stemcell Technologies; PneumaCult-ALI) at a 1:1 ratio. When a confluent monolayer was formed (2 to 4 days), the cultures were put on ALI in CBM. Cultures were differentiated for 4 weeks, and the medium was changed every 5 days.

### Virus quantification.

Growth kinetics in the primary airway cells cultured at ALI were assessed by washing the cultures twice with Dulbecco's phosphate-buffered saline (DPBS) with Ca and Mg (0.9 mM MgCl_2_ and 0.49 mM CaCl_2_), followed by HRSV inoculation from the apical side at a standardized low viral inoculum (multiplicity of infection, 0.5), based on titrations on HEp-2 cells, for 1 to 2 h at 37°C and 5% (vol/vol) CO_2_ (experiment performed once in triplicate). After inoculation, cultures were washed three times with DPBS plus Ca and Mg. At the indicated time points, supernatants from the apical side were collected by adding 200 μl Dulbecco’s modified Eagle medium (DMEM) (catalog no. LO BE12-733F; Lonza), incubating for 10 min at 37°C and 5% (vol/vol) CO_2_, and harvesting for direct titration on HEp-2 cells (39). Leftover supernatant was kept for cytokine detection. Additionally, the percentage EGFP^+^ or dTom^+^ surface area was determined by imaging complete wells by performing tile scans on an LSM700 confocal microscope, analyzed with ZEN software (Zeiss) and Fiji (40). For the analysis of fluorescence-positive area, the tile scan was selected, and Fiji was used to automatically set the optimal brightness, contrast, and threshold. Then, the software measured the percentage fluorescence area, independent of particle size (pixels).

### Histology.

Primary airway cultures were fixed in 4% (wt/vol) paraformaldehyde (PFA) for at least 30 min, after which the filters from the Transwell were excised, stored in formalin, and subsequently embedded in paraffin. Thin sections (3 μm) were prepared from the formalin-fixed, paraffin-embedded tissues and stained using hematoxylin and eosin. For IIF staining, slides were incubated at 37°C overnight and then deparaffinized (twice for 4 min in xylene, twice for 2 min in 100% ethanol, once for 2 min in 96% ethanol, once for 2 min in 90% ethanol, once for 2 min in 70% ethanol, and, lastly, 5 min of PBS). Antigens were retrieved with citrate buffer (10 mM, pH 6.0 [Sigma]) (4.2 g C_6_H_8_O_7_ · H_2_O in 2 liters Milli-Q, set pH, with 10 N NaOH). Slides were blocked in 10% normal goat serum (NGS) in phosphate-buffered saline (PBS) for 30 min at room temperature (RT). Primary antibodies (acetylated α-tubulin [Santa Cruz Biotechnology, catalog no. sc-23950 AF488] and rabbit anti-GFP [Invitrogen, catalog no. A11122]) were added for 1 h of incubation at RT and after washing secondary antibodies (acetylated α-tubulin and goat-anti-rabbit [AF594; Invitrogen, catalog no. A11012) were added for 1 h of incubation at RT. After two washings, Hoechst (Thermo Scientific, catalog no. 62249) was added and incubated for 15 min at RT. Samples were washed twice and mounted with Prolong Diamond antifade mounting medium and analyzed using an LSM700 confocal microscope, ZEN software (Zeiss), and Fiji (40).

### Immunofluorescence microscopy.

Transwell inserts were fixed in 4% (wt/vol) PFA for 30 min and then stored in PBS for further analysis. For indirect immunofluorescence staining, one-quarter of the filter was excised, washed twice, permeabilized in 0.2% Triton-X, and blocked in 10% NGS in staining buffer (DPBS with 150 mM MgCl_2_, 150 mM CaCl_2_, and 10 mM HEPES) for 30 min. The cells were incubated with conjugated mouse monoclonal antibodies for 60 min in staining buffer containing 10% NGS and 2% (wt/vol) bovine serum albumin (BSA). Tight junctions were stained using anti-zona-occludens 1 (clone 1A12; Alexa Fluor 550; Santa Cruz Biotechnologies), and cilia were stained using anti-acetylated tubulin (Alexa Fluor 647; Santa Cruz Biotechnologies, clone 6-11B-1). In the last 10 min of the incubation with antibodies, Hoechst 33342 was added (Life Technologies/Invitrogen, catalog no. 10150888). After staining, the cells were washed three times with staining buffer and mounted in Prolong antifade mounting medium (Life Technologies/Invitrogen, catalog no. 9P36961). Samples were imaged on an LSM700 confocal microscope using ZEN software (Zeiss) and Fiji (40).

### Cytokine detection.

Cytokines were measured in apical and basolateral cell culture supernatant from the primary airway cells using a human antivirus response panel (13-plex) kit (LEGENDplex; BioLegend). In short, cell culture supernatant was mixed with beads coated with capture antibodies specific for IFN-α2, IFN-γ, IFN-β, IL-28A/B, IL-29, IL-6, IL-8, IL-10, TNF-α, IL-12p70, GM-CSF, IP-10, and IL-1β and incubated for 2 h. Beads were washed and incubated with biotin-labeled detection antibodies for 1 h, followed by a final incubation with streptavidin^PE^. Beads were analyzed by flow cytometry, and final analysis was performed using the LEGENDplex analysis software v8.0. The quantity of each respective cytokine is calculated on basis of the intensity of the streptavidin^PE^ signal and a freshly prepared standard curve (as described in Weiskopf et al.) (41).

### Statistics.

The mean and standard deviation are depicted in graphs. Experiments were performed once in triplicate. Growth curves were compared by one-way analysis of variance (ANOVA) test or Student's *t* test to determine if there was statistically significant variation between groups. A *P* value of <0.05 was considered statistically significant. Pearson’s correlation coefficient was calculated to test correlation between viral loads and cytokine levels. All analyses were performed using GraphPad Prism version 8.0 for Windows.
