# Supplementary figures and images for: Human Respiratory Syncytial Virus Subgroup A and B Infections in Nasal, Bronchial, Small-Airway, and Organoid-Derived Respiratory Cultures
Source: mSphere. 2021 May 12;6(3):e00237-21. doi: 10.1128/mSphere.00237-21 (PMC8125053; doi:10.1128/mSphere.00237-21)

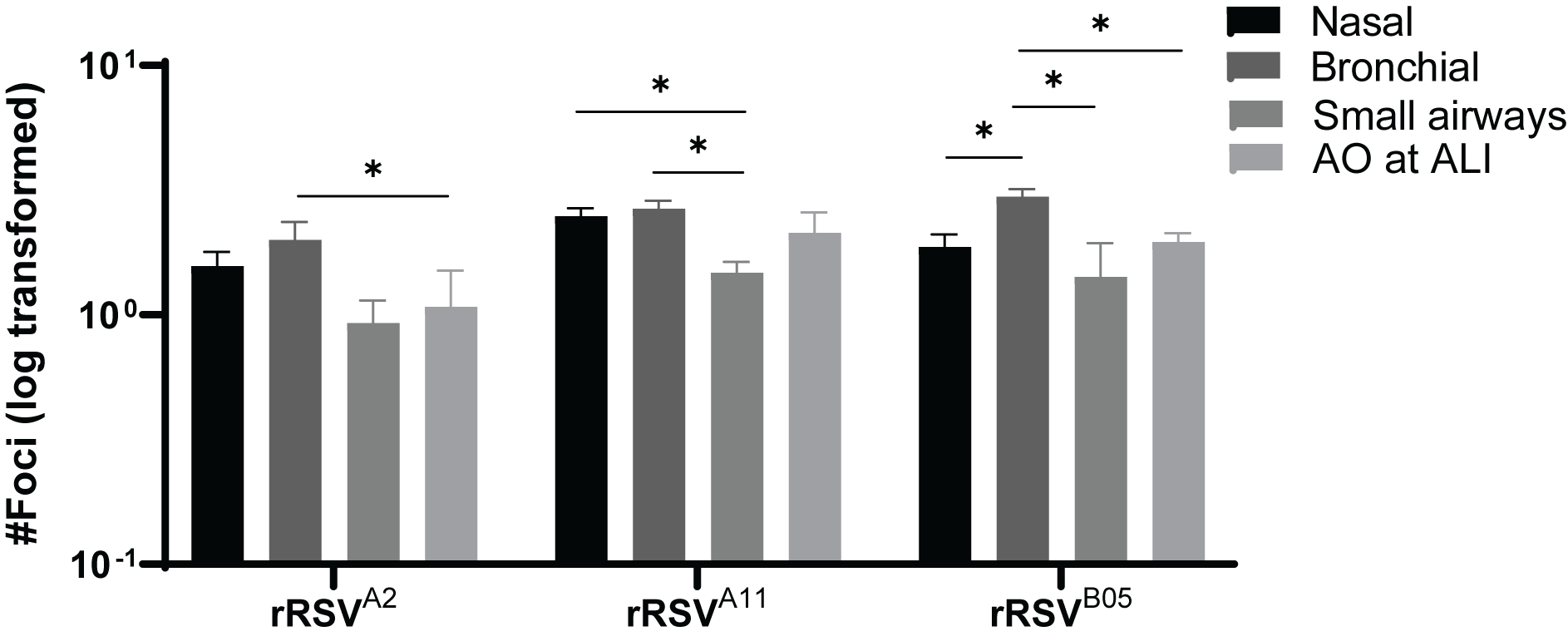

Supplement: FIG S1 [file mSphere.00237-21-sf001.tif]

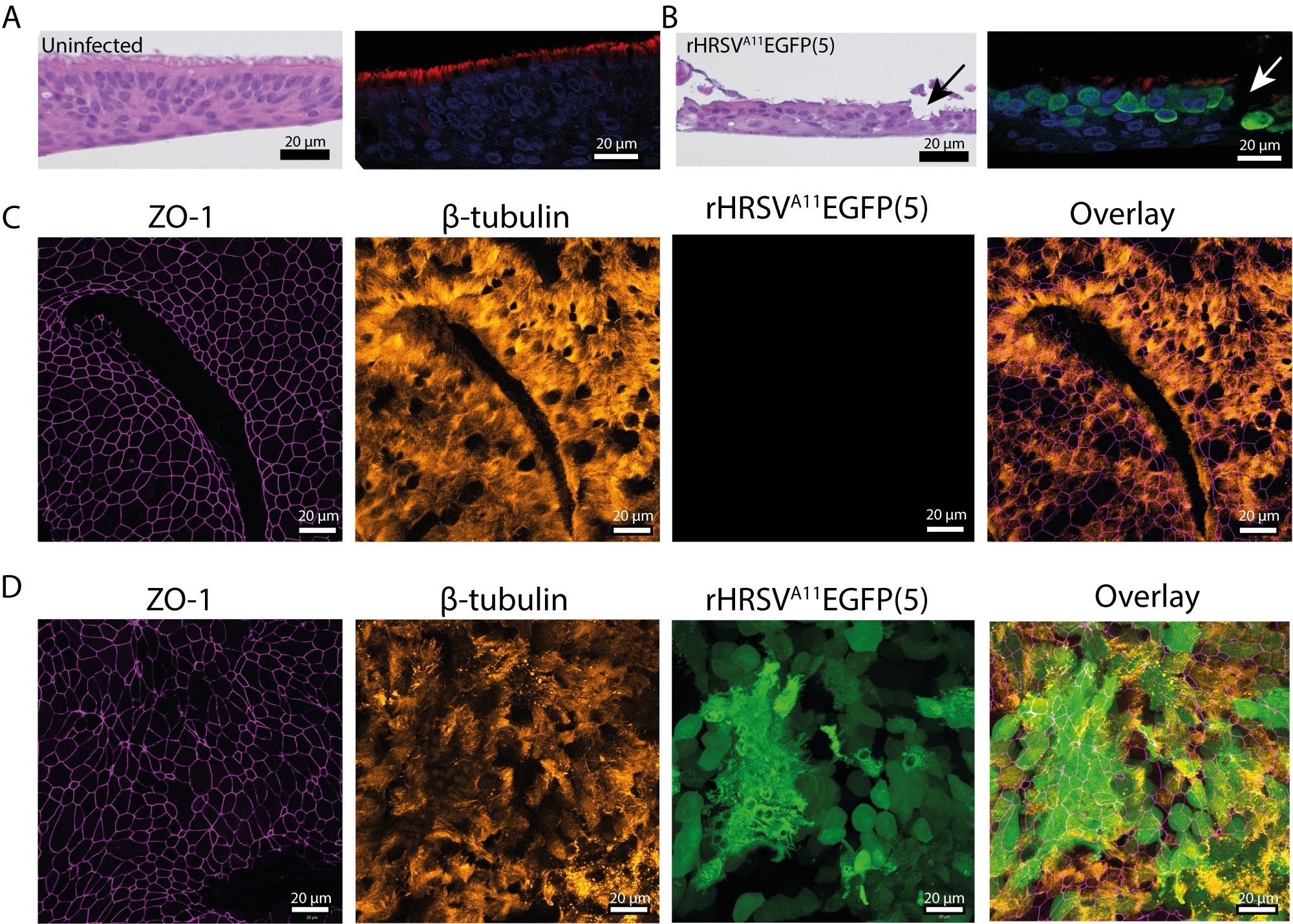

Supplement: FIG S2 [file mSphere.00237-21-sf002.tif]

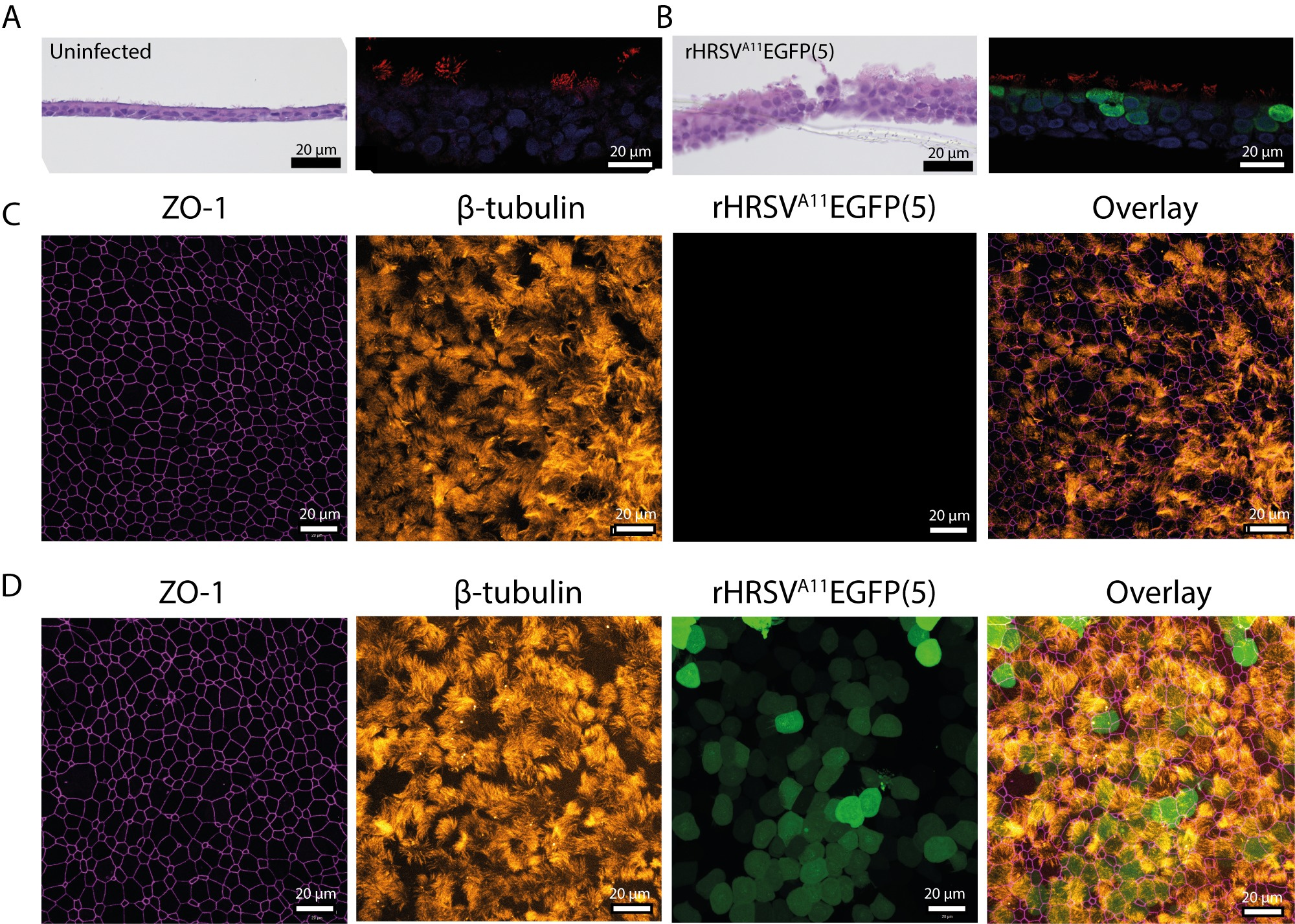

Supplement: FIG S3 [file mSphere.00237-21-sf003.tif]

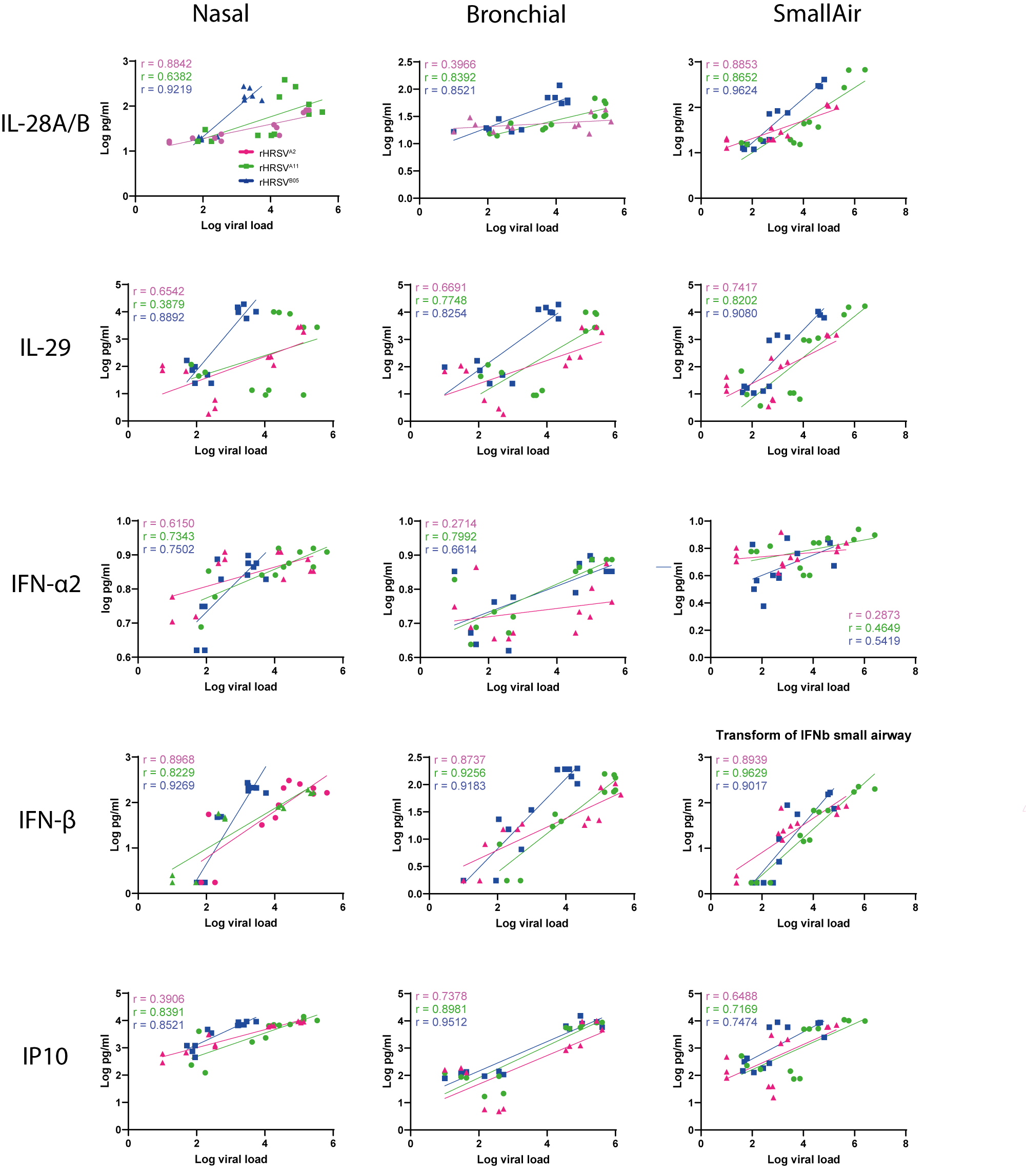

Supplement: FIG S4 [file mSphere.00237-21-sf004.tif]

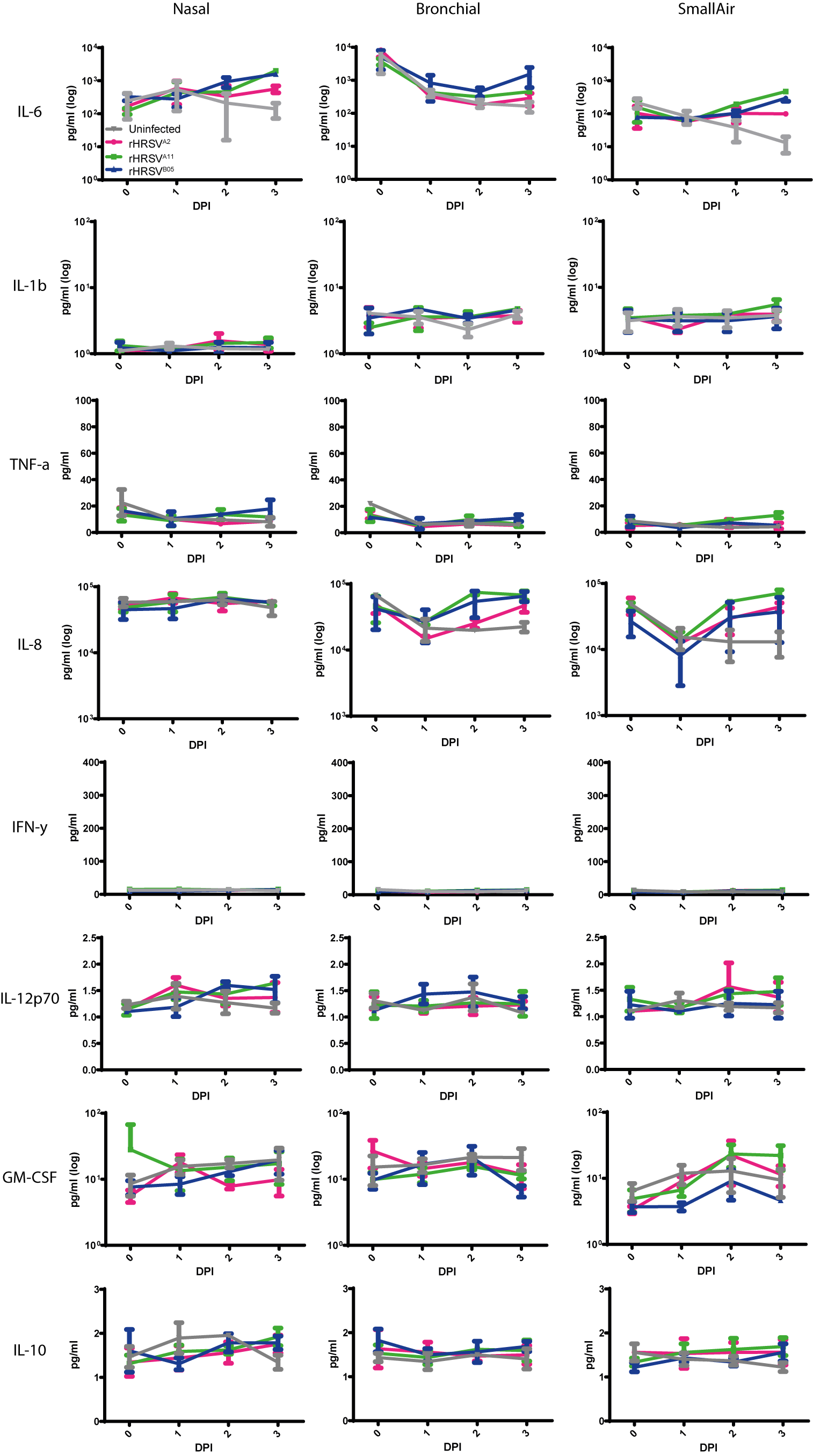

Supplement: FIG S5 [file mSphere.00237-21-sf005.tif]

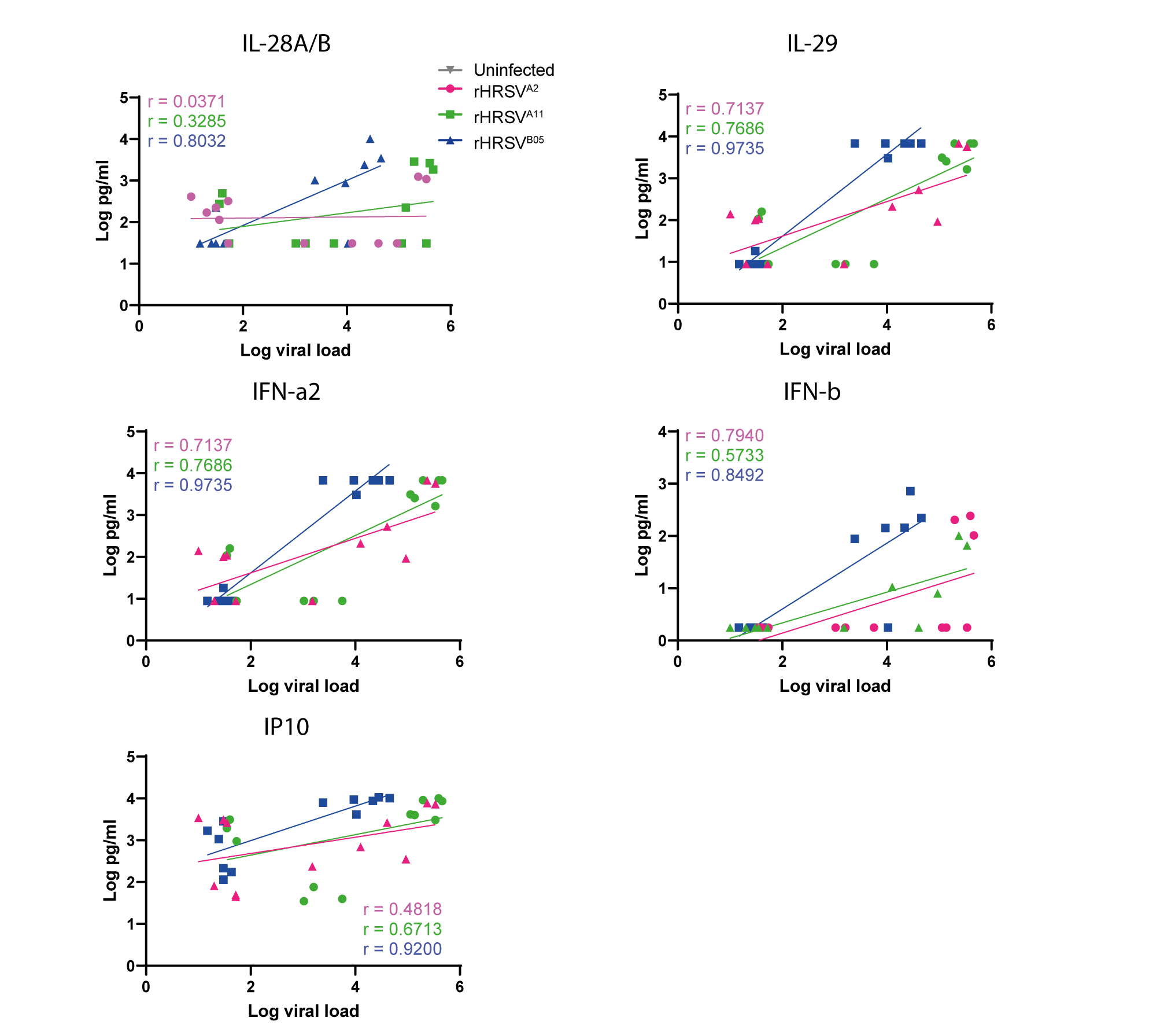

Supplement: FIG S6 [file mSphere.00237-21-sf006.tif]

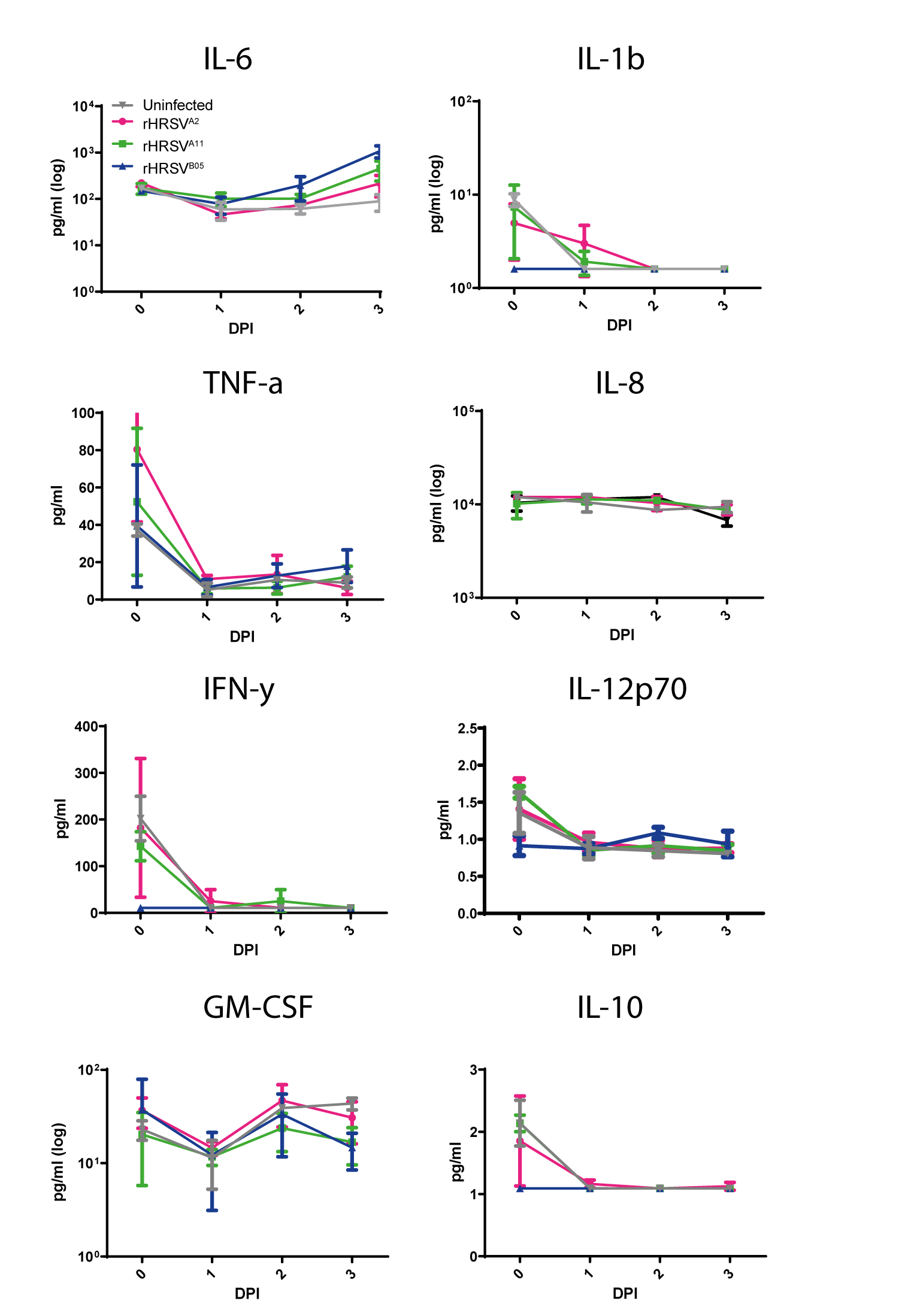

Supplement: FIG S7 [file mSphere.00237-21-sf007.tif]

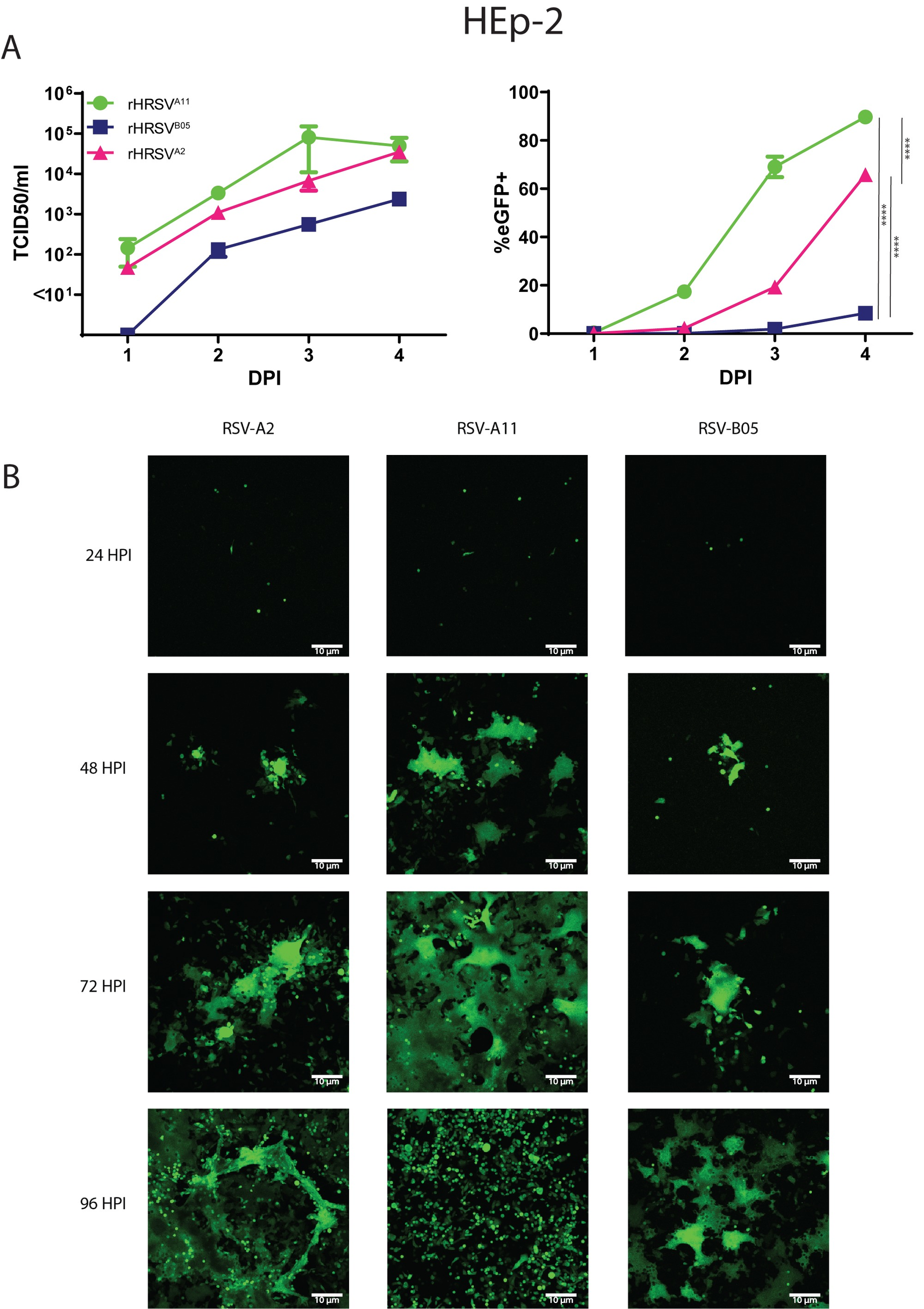

Supplement: FIG S8 [file mSphere.00237-21-sf008.tif]
